# A Miniaturized Antenna with Negative Index Metamaterial Based on Modified SRR and CLS Unit Cell for UWB Microwave Imaging Applications

**DOI:** 10.3390/ma8020392

**Published:** 2015-01-23

**Authors:** Md. Moinul Islam, Mohammad Tariqul Islam, Md. Samsuzzaman, Mohammad Rashed Iqbal Faruque, Norbahiah Misran, Mohd Fais Mansor

**Affiliations:** 1 Space Science Centre (ANGKASA), Research Centre Building, Universiti Kebangsaan Malaysia, 43600 UKM, Bangi, Selangor, Malaysia; E-Mail: rashed@ukm.edu.my; 2 Department of Electrical, Electronic and Systems Engineering, Faculty of Engineering and Built Environment, Universiti Kebangsaan Malaysia, 43600 UKM, Bangi, Selangor, Malaysia; E-Mails: tariqul@ukm.edu.my (M.T.I.); sobuzcse@eng.ukm.my (M.S.); bahiah@eng.ukm.my (N.M.); fais@eng.ukm.my (M.F.M.)

**Keywords:** microwave imaging, metamaterial, negative index, UWB

## Abstract

A miniaturized antenna employing a negative index metamaterial with modified split-ring resonator (SRR) and capacitance-loaded strip (CLS) unit cells is presented for Ultra wideband (UWB) microwave imaging applications. Four left-handed (LH) metamaterial (MTM) unit cells are located along one axis of the antenna as the radiating element. Each left-handed metamaterial unit cell combines a modified split-ring resonator (SRR) with a capacitance-loaded strip (CLS) to obtain a design architecture that simultaneously exhibits both negative permittivity and negative permeability, which ensures a stable negative refractive index to improve the antenna performance for microwave imaging. The antenna structure, with dimension of 16 × 21 × 1.6 mm^3^, is printed on a low dielectric FR4 material with a slotted ground plane and a microstrip feed. The measured reflection coefficient demonstrates that this antenna attains 114.5% bandwidth covering the frequency band of 3.4–12.5 GHz for a voltage standing wave ratio of less than 2 with a maximum gain of 5.16 dBi at 10.15 GHz. There is a stable harmony between the simulated and measured results that indicate improved nearly omni-directional radiation characteristics within the operational frequency band. The stable surface current distribution, negative refractive index characteristic, considerable gain and radiation properties make this proposed negative index metamaterial antenna optimal for UWB microwave imaging applications.

## 1. Introduction

Microwave imaging systems have recently been used extensively for medical imaging applications. Usually, these imaging systems are constructed with a circular cylindrical array antenna and used to detect cancerous tissue. These systems have gradually attracted great interest in medical applications. The ultra-wideband signal provides good resolution and penetration properties. The use of such microwave imaging systems has been proposed to detect breast cancer [1,2,3]. In these studies, compact directional ultra-wideband antennas are used to transmit and receive short duration pulses that are directed into the breast tissues. The tumor detection capability originates from the considerable contrast between the electrical properties (conductivity and permittivity) of normal and cancerous tissues. A tumor causes the scattering of an electromagnetic wave as a reflecting object due to the differences in those properties. A negative-index metamaterial is a left-handed metamaterial that contains an engineered electromagnetic structure with some extraordinary properties such as negative permittivity, and negative permeability, as well as a negative refractive index over a specific frequency band that is not usually found in nature. Metamaterials have created a new era in microwave imaging applications because of their great potentials for the production of effective microwave devices, such as antennas. Veselago made the first theoretical speculation of the existence of a material that could exhibit negative permeability and negative permittivity simultaneously [4]. Then, Pendry constructed metamaterials with the help of the split ring resonator (SRR) where the electromagnetic (EM) wave is conducted via a route that opposes the convectional path [5]. Finally, in 2000, Smith successfully exhibited and validated this concept by constructing a new artificial material known as a left-handed metamaterial in which both ɛ and µ were negative [6]. Different types of LHMs have been proposed using various structures such as SRRs [7], spiral resonators [8], fishnet structures [9], double-sided SRRs [10], double-bowknot shaped resonators [11], transmission-line based structures [12], periodic arrays of H-shaped pairs [13], SRR pairs [14], cut wire pairs [15], broad side coupled SRRs [16] and complementary electric field-coupled resonator (CLEC) [17]. The field of LHM research has been expanded by adopting various techniques. These studies face difficulties such as their narrow bandwidth, which limits the range and spectrum of their applications. They also have limited utility in antenna design and fabrication, as these materials are difficult to fabricate and use. Therefore, there is a rise in the demand for research to overcome these difficulties and broaden the fields of metamaterial applications.

A left-handed metamaterial structure is proposed to increase the gain of a microstrip antenna [18]. The performance of the antenna was studied by placing the LHM structure in front of the patch antenna. The results demonstrate that this antenna structures exhibits higher gain and a greater directional characteristic because of the placement of the LHM. However, this design technique leads to larger antenna dimensions. An elliptical tapered slot antenna of 50 mm × 50 mm is proposed for UWB medical imaging systems [19]. A compact metamaterial antenna is presented for UWB applications covering from the 5.2–13.9 GHz frequency band, where the optimum gain is 1.2–3.85 dBi and the directivity is 1.95–5.45 dB [20]. The results demonstrate that the reported metamaterial is effectively applicable to the production of materials with negative indices at low cost. However, the resulting antenna dimensions are large, gain and directivity are small, and the reported metamaterial antenna does not completely cover the UWB band. TEM horn antennas based on aperture raster scanning have been reported for near-field microwave imaging [21]. The horn antenna features a high gain and excellent decoupling from the outside environment. However, the large sizes of the antenna and high cost due to its fabrication complexity must be considered. A compact metamaterial antenna was reported using two transmission lines metamaterial arms [22]. Each metamaterial arm consists of a microstrip transmission-line loaded with five spiral inductors. The reported antenna delivers a bandwidth of 100 MHz with a radiation efficiency of 65.8% at 3.30 GHz. A microstrip-fed Dark Eyes antenna of 22.25 mm × 20 mm was designed for near-field microwave sensing [23]. A compact UWB metamaterial antenna was proposed that used a modified split-ring resonator (SRR) and capacitively loaded strips (CLS) [24]. Three unit cells were used as the radiating element. This antenna provide 2.9–9.9 GHz bandwidth (below −10 dB) not completely covering the UWB band. A resistively loaded ultra-compact broadband antenna was designed for microwave breast cancer detection [25]. Larger dimensions were considered while operating in the same frequency band. A microstrip antenna that employed left-handed metamaterials in conjunction with one dipole and six LHM unit cells was presented [26]. The antenna provides 3 dB directivity and −1 dBi maximum gain, along with radiation efficiency of 40% at 2.50 GHz. Several UWB antenna designs with different shapes, low distortion and compact size have been proposed for use in microwave imaging systems [27,28,29]. Each has its own merits and drawbacks. Some of the proposed antennas lack a planar structure, whereas others have low-gain and/or low radiation efficiency. Electrically small antenna has been explained to improve overall performance where near-field resonant parasitic (NFRP) Egyptian axe dipole elements are used [30].

In this research, a negative index metamaterial antenna with modified SRR and CLS that attains a compact UWB profile omni-directional radiation characteristics, favorable gain and reasonable current distribution is presented. The described metamaterial antenna consists of four left-handed (LH) metamaterial (MTM) unit cells with a partial ground plane containing a rectangular slot on the upper portion, generating an ultra-wide bandwidth ranging from 3.40 to 12.5 GHz. The antenna formation is smooth with simple design and comfortable fabrication. Metamaterial unit cells are installed on the radiating patch with a modified SRR and a CLS to obtain design architecture that simultaneously exhibits both negative permittivity and negative permeability, a stable negative refractive index to improve the antenna performance for microwave imaging.

## 2. The Metamaterial Unit Cell Configuration

**Figure 1:**
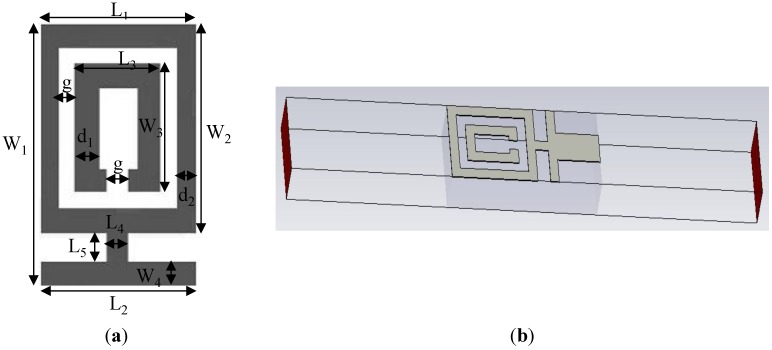
(**a**) Front view of the modified SSR unit cell with CLS and (**b**) simulation geometry.

The proposed metamaterial antenna design starts with a metamaterial unit cell for UWB microwave imaging application. The unit cell is designed with its resonance within the UWB range of 3.1–10.6 GHz. There are well-known methods of metamaterial design to provide simultaneous negative permittivity and permeability using SRRs [5,6]. The SRR is constructed using two loops that are structured as two opposing concentric split rings [5]. The SRR is a magnetically resonant structure that leads to a perpendicular magnetic field whose application generates negative permeability. A split gap is added to the inner ring, allowing capacitance to be introduced, which also controls the resonant properties of the structure. Figure 1a shows the front view of the modified SRR with a CLS structure. The proposed design is modified by the closure of the outer ring. This modification decreases the series capacitance of the SRR structure and increases the coupling between the inner and outer rings. This metamaterial unit cell is printed on FR4 low dielectric substrate material with a thickness of 1.6 mm and a dielectric constant of 4.6. A CLS is added to the modified SRR metamaterial unit cell, so that the resonance property is achieved within the operating UWB range. A CLS is an I-shaped strip line that acts as an electric dipoles and mimics a long metallic wires [20]. The combination of SRR and CLS permits simultaneous magnetic and electric resonance due to the SRR resonance through a perpendicular magnetic field and the CLS resonance through a parallel electric field [31]. A lower resonance is enabled through the two resonances for the entire structure, aided by the combined induced current [32]. The design parameters of the metamaterial unit cell are listed in Table 1.

**Table 1:** Design parameters of the metamaterial unit cell.

Design parameter	Dimension (mm)	Design parameter	Dimension (mm)
Length of CLS, L_2_	3.2	Width of the inner SRR, W_3_	2.4
Width of CLS, W_4_	0.4	Split gap within inner SRR, g	0.4
Width of the distance between outer SRR and CLS, L_5_	0.6	Space between inner and outer SRR, g	0.4
Length of the distance between outer SRR and CLS, L_4_	0.4	Width of inner SRR line, d_1_	0.4
Length of the outer SRR, L_1_	3.2	Width of outer SRR line, d_2_	0.4
Width of the outer SRR, W_2_	4.0	Width of combined SRR and CLS, W_1_	5.0
Length of the inner SRR, L_3_	1.6	-	-

**Figure 2:**
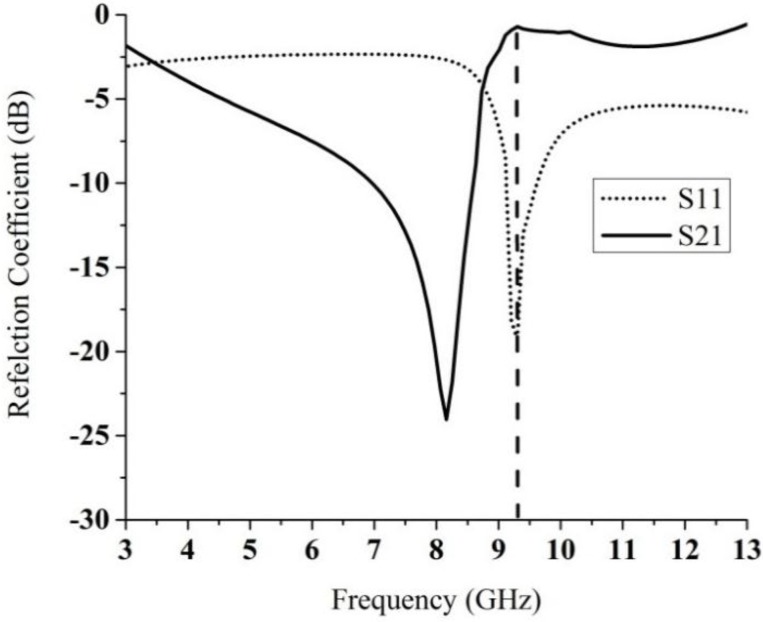
Simulation results of S-parameters for the unit cell plotted in Figure 1.

In this study, the LHM is executed using the finite-difference time domain (FTTD) based on computer simulation technology (CST) software to achieve the S-parameters the reflection coefficient (S_11_) and transmission coefficient (S_21_). Figure 1b illustrates the simulation setup for the unit cell of the LHM. The structure that is to be tested has been placed between two waveguide ports on the positive and negative x-axis and is excited by an electromagnetic wave in the direction of the x-axis. A perfect electric conductor (PEC) boundary condition has been defined along the walls perpendicular to the y axes, and the walls perpendicular to z-axes are defined to be perfect magnetic conductor boundaries, the simulation arrangement is displayed in Figure 1b. The incident wave propagates in the x-axis direction, while the E-field of the incident wave is polarized along the y- axis, and the H-field of the incident wave is polarized along the z-axis. For the simulation, a frequency domain solver was used. The normalized impedance was set to 50 Ω. The simulation was performed over the frequency range of 2–16 GHz. The S parameters that were obtained from the simulation were exported to Math CAD software. Figure 2 shows that a transmission peak occurs at a frequency of 9.4 GHz, which denotes a left-handed band. The principal augmentation is the enhanced production of the proposed metamaterial magnetic response from the larger overall current, self-resonance and overlapping responses in comparison with conventional SRRs designs. To verify the electromagnetic characteristic of the proposed left-handed metamaterials, the retrieval algorithm provided in [33,34] is applied to achieve the constitutive effective parameters depending on the transmission and reflection coefficient properties. These equations are applied individually.

(1) $$z=\sqrt{\frac{{\left(1+S_{11}\right)}^{2}-S_{21}^{2}}{{\left(1-S_{11}\right)}^{2}-S_{21}^{2}}}$$

(2) $$e^{\left(jnk_{0}d\right)}=A\pm j\sqrt{\left(1-A^{2}\right)}$$

(3) $$A=\frac{\left(1-S_{11}^{2}+S_{21}^{2}\right)}{\left(2S_{21}\right)}$$

(4) ε=n/z

(5) μ=n×z

where, *z*, the impedance; ε, the relative effective permittivity; μ, the permeability; *n*, the refractive index; *k*_0_, the wavenumber of the incident wave in the free space; *d*, the slab thickness of the metamaterial.

**Figure 3:**
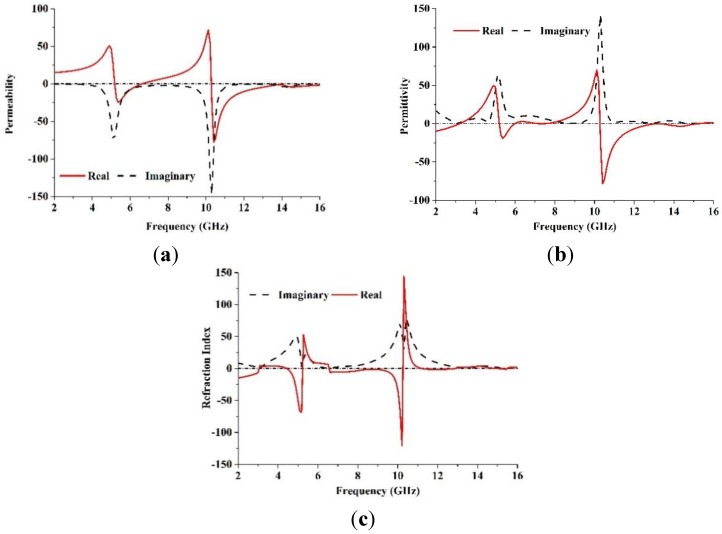
The observed effective parameters such as (**a**) permeability (**b**) permittivity (**c**) refractive index of the proposed unit cell.

To retrieve the effective parameters, Equations (1)–(5) provided above are used. Figure 3 shows the retrieved effective parameters, such as the permeability, permittivity and refractive index of the proposed LHM unit cell. Table 2 summarizes the details of the negative refractive index frequency band. It can be seen from Table 2 that the LHM unit cell has a different resonance bandwidth in the negative refractive index frequency regions. This behavior indicates improved effective of LHM structures parameters compared to the LHMs described in [9,10,12,15,16,20] enabling negative values over a broad band frequency.

**Table 2:** Negative index frequency region of the retrieved effective parameters.

Parameter	Negative index frequency region (GHz)
Permeability, µ	5.28–6.61, 10.31–13.92
Permittivity, ɛ	5.3–6, 7.27–7.37, 10.31–13.26
Refractive index, *n*	4.52–5.18, 6.61–10.22, 11.26–12.78

## 3. The MTM Antenna

**Figure 4:**
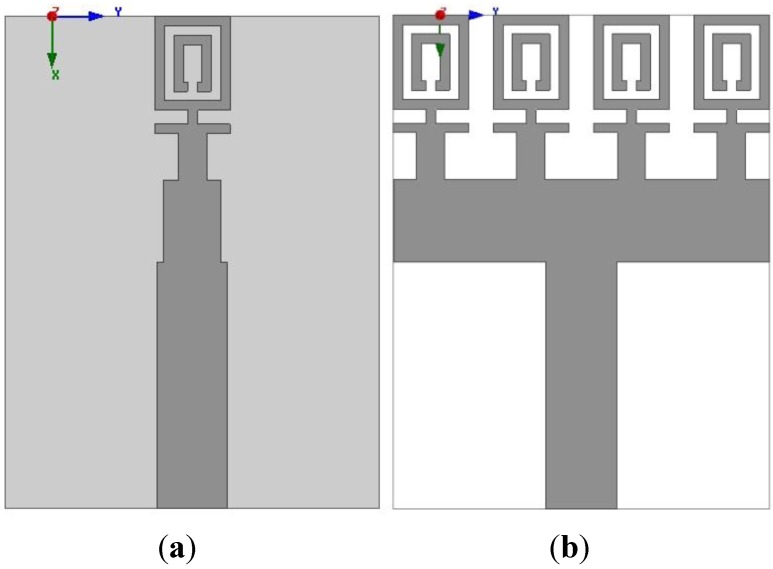
(**a**) One element MTM antenna and (**b**) four-element MTM antenna.

**Figure 5:**
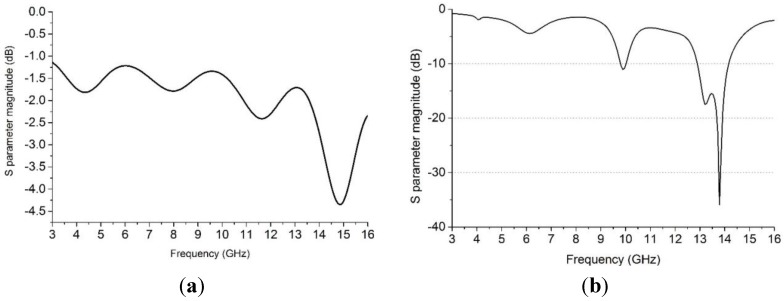
The reflection coefficient, S_11_, of the MTM antenna (**a**) one element (**b**) four elements.

The structural design of the proposed metamaterial UWB antenna starts with the use of one unit cell as the radiating element. Figure 4 illustrates the MTM antenna with one element and four elements. The dimensions of the MTM antenna are 16 mm × 21 mm. An impedance of 50 Ω is provided by the port. The MTM antenna structure is simulated using the EM solver Computer Simulation Technology (CST). The reflection coefficient of the MTM is plotted in Figure 5 with one element and four elements. It can be observed from Figure 5 that the antenna is better matched at the higher resonance frequencies in the cases of both one element and four elements. The objective is to obtain an UWB frequency range for a MTM antenna with negative index metamaterial characteristics to be used in microwave imaging.

## 4. UWB Metamaterial Antenna and Microwave Imaging

**Figure 6:**
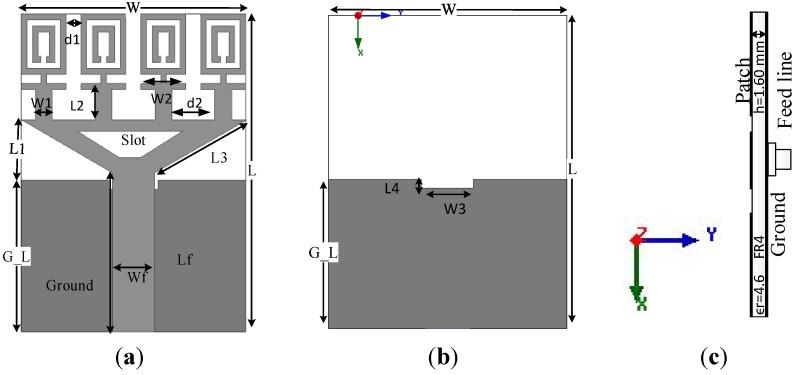
Proposed antenna (**a**) geometry layout (**b**) bottom view (**c**) Cross sectional view.

The outline of the proposed negative index UWB metamaterial antenna is illustrated in Figure 6. This antenna is based on FR4 substrate material with 1.6 mm thickness and a dielectric constant of 4.6. The geometric parameters of the antenna are identified after post-optimization and they are listed in Table 3. This antenna consists of a radiating patch of four unit cells placed periodically along the y-axis, a microstrip trident-shaped feeding strip and a partial ground plane. Every unit cell is identical to the others. The improved bandwidth and radiation characteristics of the proposed MTM antenna are achieved using these design techniques.

**Figure 7:**
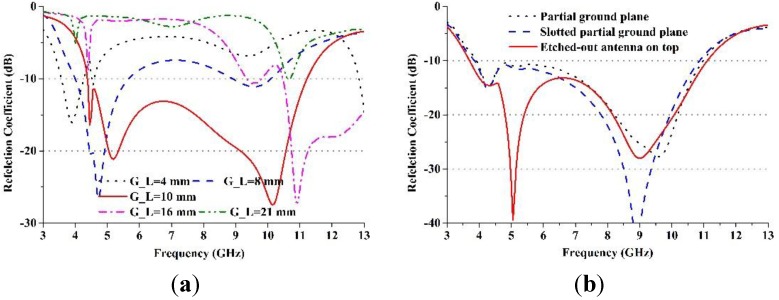
(**a**) The effect of the size of the ground plane, G_L, on the reflection coefficient; (**b**) comparisons between the reflection coefficient of the partial ground plane, G_L = 10 mm, slotted partial ground plane, and etched-out antenna on top of the slotted partial ground plane.

Figure 7a demonstrates the effect of the size of the ground plane, G_L, on the reflection coefficient. It was found that the best simulation results could be achieved for the proposed negative index metamaterial antenna in terms of the reflection coefficient, where G_L = 10 mm within the operating UWB frequency band. Figure 7b illustrates the comparisons between the reflection coefficient of the partial ground plane, G_L = 10 mm, slotted partial ground plane, and etched-out antenna on top of the slotted partial ground plane. It can be observed from Figure 7b that the proposed metamaterial antenna provides the optimum results with an etched-out antenna on top with respect to the reflection coefficient.

**Table 3:** Antenna design specifications (according to Figure 6).

Parameter	Dimension (mm)	Parameter	Dimension (mm)
W	16	G_L	10
L	21	L3	7.37
d1	1.06	Wf	3
W1	1.2	Lf	10.5
L2	2	L4	0.6
d2	3.07	W3	3.5
L1	4	-	-

**Figure 8:**
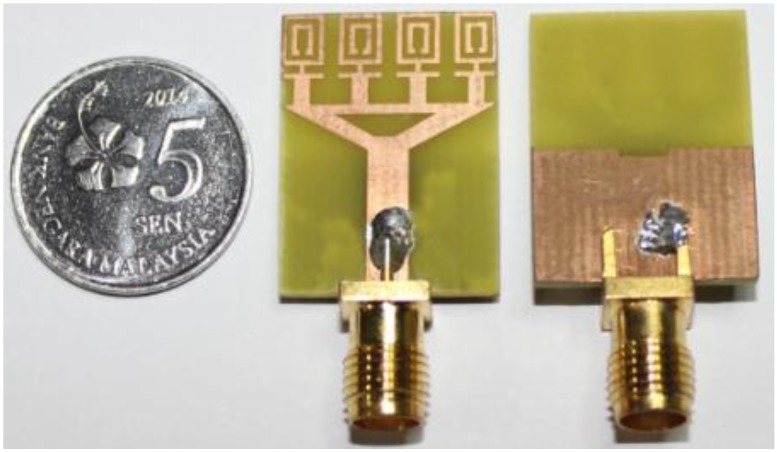
Fabricated prototype of the proposed negative index UWB MTM antenna.

**Figure 9:**
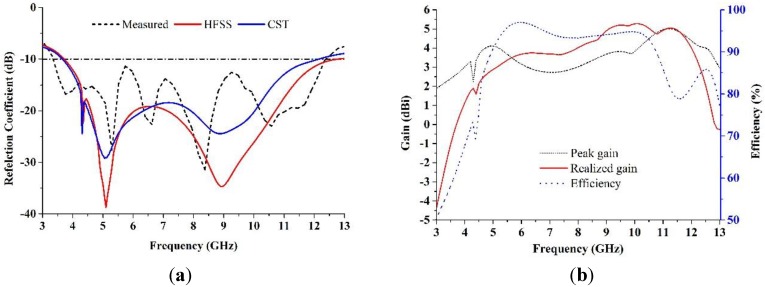
(**a**) Comparison between the simulated and measured reflection coefficient and (**b**) measured gain and radiation efficiency of the proposed UWB MTM antenna.

The proposed negative index metamaterial antenna was fabricated and measured to verify the ultra-wideband result by applying an Agilent Technologies N5230A PNA-L Network Analyzer at the Microwave Laboratory, Space Science Centre (ANGKASA), UKM, Malaysia. The fabricated antenna is shown in Figure 8. Figure 9a shows the validation of the ultra-wide band performance. The measured reflection coefficients were compared with the simulation results using CST and HFSS, and a frequency band of 3.4–12.5 GHz was identified from the measurements for a voltage standing wave ratio of less than 2. The dissimilarities between the measured and simulated antenna results are likely due to fabrication and soldering faults. Figure 9b shows the measured gain and radiation efficiency of the proposed UWB MTM antenna. It can be found from Figure 9b that the radiation efficiency is 88%, the average gain is 3.95 dBi and the maximum gain is 5.16 dBi.

**Figure 10:**
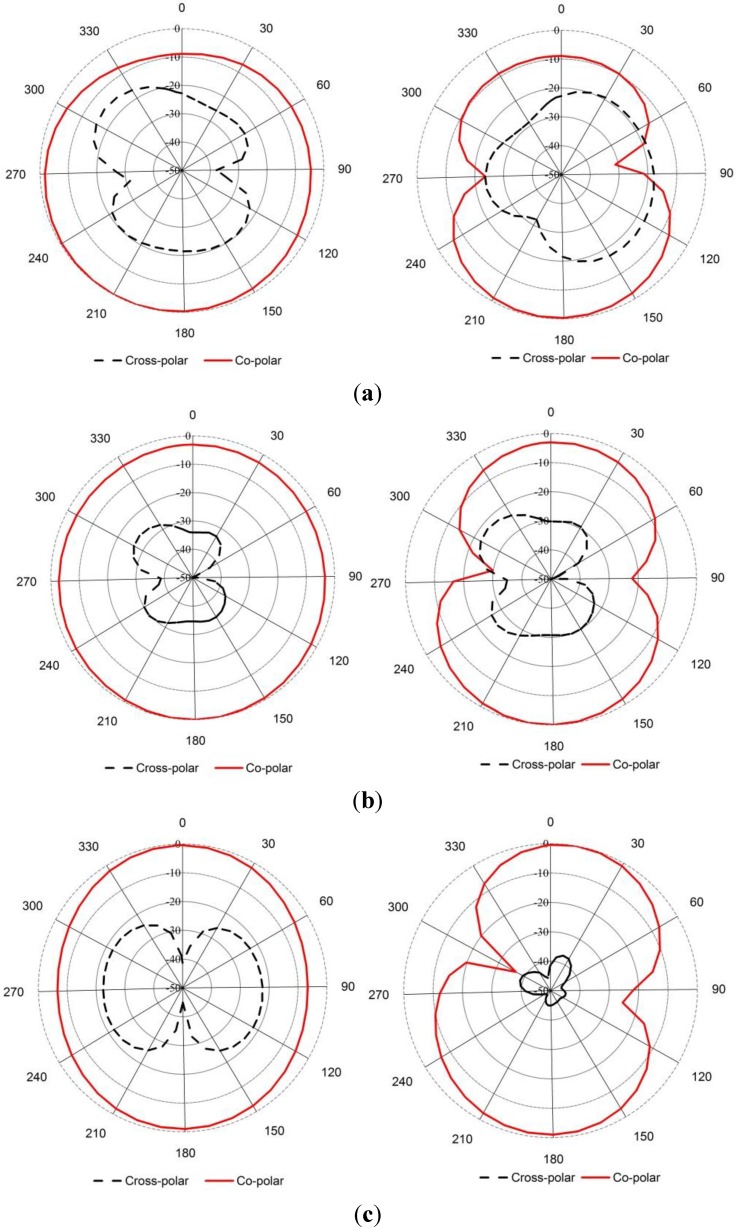

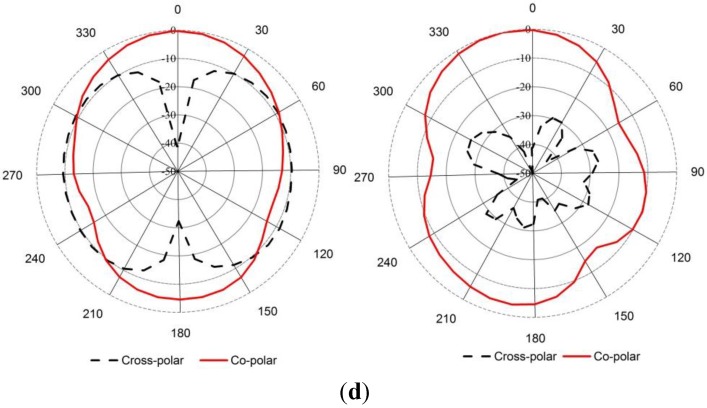
Measured radiation pattern of the proposed antenna at (**a**) 4 GHz; (**b**) 6 GHz; (**c**) 9 GHz and (**d**) 12 GHz.

**Figure 11:**
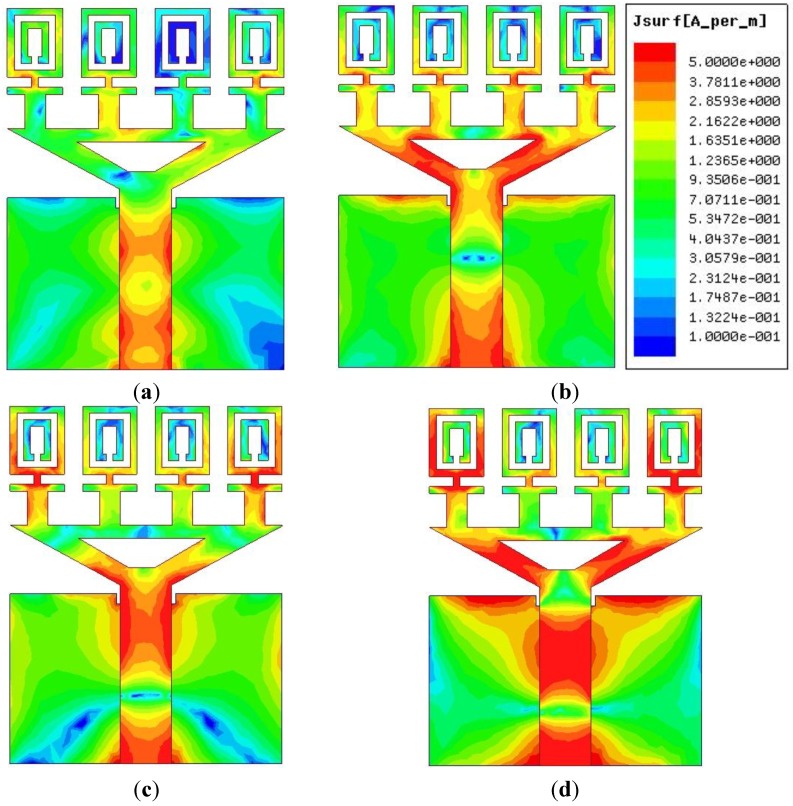
The surface current distribution at (**a**) 4 GHz; (**b**) 6 GHz; (**c**) 9 GHz and (**d**) 12 GHz.

The measured radiation pattern of the proposed negative index UWB metamaterial antenna is plotted in Figure 10 at 4 GHz, 6 GHz, 9 GHz and 12 GHz in the xz plane (E-plane) and yz plane (H-plane). It can be observed from Figure 10 that a nearly omni-directional radiation pattern exists over the 3.4–12.5 GHz frequency range. The cross polarization level is lower than the co-polarization level, which is the desired result. The surface current distributions of the proposed UWB negative index metamaterial are demonstrated in Figure 11 at 4 GHz, 6 GHz, 9 GHz and 12 GHz. It can be observed from Figure 11 that the currents are flowing dominantly along the x-axis. This flow indicates an omni-directional antenna attitude. It can also be observed that the flow of currents is strong at the metamaterial unit cell because of its negative index frequency band. To ensure an equitable comparison between the proposed antenna and the antenna design studied [18,19,22,23,26,27,28] (all reference antennas cover the UWB spectrum); their performances parameters, such as the 10-dB bandwidth, dimensions, electrical dimensions, fractal bandwidth and gain are discussed. Although the proposed antenna may not have a better gain than of the references [18,27,28], a good FBW (114.50%) with a smaller electrical dimension is exhibited. Therefore, the proposed UWB metamaterial antenna can offer good compact characteristics while maintaining much smaller dimensions than the designs in [18,19,22,23,26,27,28]. Table 4 summarizes the existing antennas with the proposed UWB metamaterial antenna.

**Table 4:** Comparison of existing antennas with the proposed UWB metamaterial antenna.

Antennas	Application	BW GHz (−10 dB)	Dimension (mm^2^)	Electrical dimension	FBW (%)	Gain dBi
[19]	Medical Imaging	3.10–11.00	50 × 50	0.52 λ × 0.52 λ	112.01	4.3~10.8
[20]	Ultra-Wideband	5.20–13.90	25 × 25	0.43 λ × 0.43 λ	91.01	1.2~3.85
[23]	Microwave Sensing	2.70–9.70	22.25 × 20	0.20 λ × 0.18 λ	112.90	not reported
[24]	Ultra-Wideband	2.90–9.90	22 × 21	0.21 λ × 0.20 λ	109.38	−1.0~5.0
[27]	Microwave Imaging	3.80–11.85	30 × 30	0.38 λ × 0.38 λ	102.00	not reported
[28]	Microwave Imaging	1.15–4.40	75 × 75	0.29 λ × 0.29 λ	117.12	2.0~8.0
[29]	Microwave Imaging	4.0–9.0	30 × 30	0.40 λ × 0.40 λ	76.92	2.0~6.0
Proposed	Microwave Imaging	3.40–12.50	16 × 21	0.18 λ × 0.24 λ	114.50	1.0~5.16

**Figure 12:**
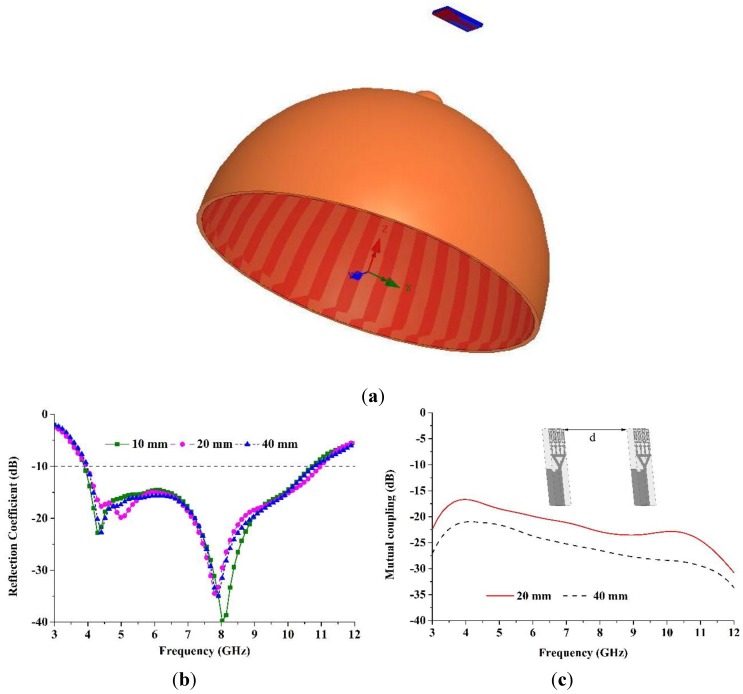
(**a**) Simulation model depicting the breast and the proposed metamaterial antenna; (**b**) reflection coefficient variation with frequency from the breast at various distances; (**c**) mutual coupling variation between two similar antennas with respect to frequency at two different lengths.

Figure 12a illustrates the simulation model including the breast and the proposed metamaterial antenna. In the microwave imaging system, the proposed UWB antenna is kept the breast phantom for breast cancer detection simulation. Then, the effects of the breast tissues are studied on the performance of the antenna. To simulate a breast phantom containing two layers, *i.e.*, the skin layer and the breast tissue layer, the electromagnetic model is utilized. The skin layer has the following properties: dielectric constant = 38, thickness = 2.5 mm, and conductivity = 1.49 S/m. The breast tissue layer has a maximum width of 8.75 cm, with conductivity = 0.141 S/m and dielectric constant = 5.14. Figure 12b plots the results of the simulation between the antenna and the breast for different distances such as 10 mm, 20 mm, and 40 mm. It can be observed clearly from Figure 12b that this proposed antenna shows its ultra wideband characteristic, even when it is very near the breast tissues.

The proposed antenna is applied to a microwave imaging system that holds an array of antennas enclosing the breast phantom. Nevertheless, it is essential to examine the value of the mutual coupling between those antennas. The mutual coupling was calculated between two similar antennas at various frequencies, taking two values, (20 mm and 40 mm) for the distance between them, as shown in Figure 12c. The two antennas were mutually coupled and parallel to each other. Figure 12c plots the outcomes of this calculation. It can be observed that the mutual coupling is below 17 dB over the operating bands when the distance is 20 mm between the two antennas and 21 dB when the distance is 40 mm between the two antennas. Figure 12c shows that the coupling increases as the distance falls between the two similar antennas.

The correlation coefficient between the transmitted signal and the received signal illustrates the amount of pulse distortion that is induced by the antenna. The fidelity factor (F) is defined using Equation (6) described in [35]:

(6) $$F={\text{max}}_{\tau }\left|\frac{\int_{-\infty }^{+\infty }s(t)r(t-\tau )}{\sqrt{\int_{-\infty }^{+\infty }s{\left(t\right)}^{2}dt.\int_{-\infty }^{+\infty }r{\left(t\right)}^{2}dt}}\right|$$

where *s* (*t*) and *r* (*t*) are the transmitted and received signals, respectively. To avoid losing the modulated information, a high degree of correlation between the transmitted and received signals is necessary for impulse radio in UWB communications. However, the fidelity factor is not compulsory for most other telecommunication systems. The time domain characteristics of this proposed UWB antenna were also determined. Two configurations as face-to-face and side-by-side orientations were chosen.

**Figure 13:**
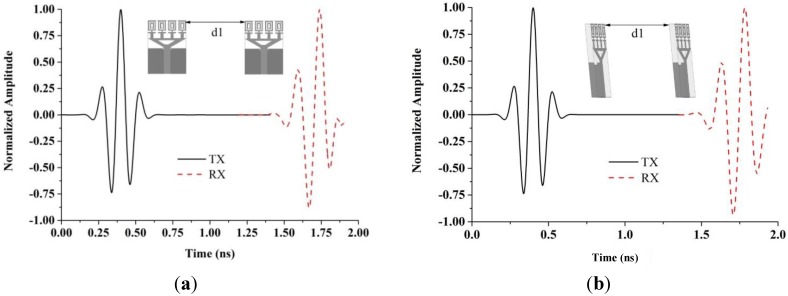
Transmitted and received pulses (**a**) side by side and (**b**) face to face.

A narrow pulse was sent from the broadcasting antenna and the received pulse was calculated at the receiving antenna at a distance d1 of 300 mm from the sender. Figure 13 illustrates the shapes of the received and transmitted pulses. The received pulse and the transmitted pulse were normalized by their maximum levels. This graph demonstrates the negligible pulse distortion with respect to the peak value of 1. The fidelity factor is 0.87 for the face-to-face and 0.79 for the side-by-side configurations. As a result, the proposed antenna supports a narrow distortion less pulse for operation.

## 5. Conclusions

A miniaturized UWB metamaterial antenna with a negative index characteristic for use in microwave imaging applications has been proposed. The complete design technique is described for the negative index unit cells and the antenna. The fabricated negative index metamaterial antenna provides 114.5% bandwidth covering the frequency band of 3.4–12.5 GHz for a voltage standing wave ratio of less than 2 with a maximum gain of 5.16 dBi at 10.15 GHz. The overall antenna dimensions are 16 mm × 21 mm × 1.6 mm. From the simulated results, it can be shown that the proposed metamaterial antenna exhibits UWB characteristic when it is very close to the breast phantom with only a small distortion of the time domain characteristics. Mutual coupling was simulated between two closely positioned similar UWB antennas, and low mutual coupling was observed. The directive gain, low mutual coupling, considerable VSWR, negative refractive index characteristic, and stable surface current distribution ensure that the proposed antenna is a promising candidate for UWB microwave breast cancer imaging applications.
